# Shape-morphing composites with designed micro-architectures

**DOI:** 10.1038/srep27933

**Published:** 2016-06-15

**Authors:** Jennifer N. Rodriguez, Cheng Zhu, Eric B. Duoss, Thomas S. Wilson, Christopher M. Spadaccini, James P. Lewicki

**Affiliations:** 1 Lawrence Livermore National Laboratory, 7000 East Avenue, Livermore, California, 94550, USA

## Abstract

Shape memory polymers (SMPs) are attractive materials due to their unique mechanical properties, including high deformation capacity and shape recovery. SMPs are easier to process, lightweight, and inexpensive compared to their metallic counterparts, shape memory alloys. However, SMPs are limited to relatively small form factors due to their low recovery stresses. Lightweight, micro-architected composite SMPs may overcome these size limitations and offer the ability to combine functional properties (e.g., electrical conductivity) with shape memory behavior. Fabrication of 3D SMP thermoset structures via traditional manufacturing methods is challenging, especially for designs that are composed of multiple materials within porous microarchitectures designed for specific shape change strategies, e.g. sequential shape recovery. We report thermoset SMP composite inks containing some materials from renewable resources that can be 3D printed into complex, multi-material architectures that exhibit programmable shape changes with temperature and time. Through addition of fiber-based fillers, we demonstrate printing of electrically conductive SMPs where multiple shape states may induce functional changes in a device and that shape changes can be actuated via heating of printed composites. The ability of SMPs to recover their original shapes will be advantageous for a broad range of applications, including medical, aerospace, and robotic devices.

Recently, there has been an increase in demand for the conversion of renewable and nontoxic biomass into polymeric materials^1^,^2^,^3^,^4^. In particular, bio-based SMPs are attractive due to their potential biodegradability and biocompatibility, while being derived from inexpensive and abundant renewable resources. Shape memory polymers (SMPs) are a category of smart materials^5^,^6^ that have the capability to recover their permanent shapes from one or multiple temporarily deformed states when exposed to an external stimulus^7^, such as temperature^8^, moisture^9^, light^10^, electromagnetic field^11^, or ∆pH^12^. Specifically, thermally actuated SMPs have found wide applications in biomedical devices^13^,^14^,^15^,^16^,^17^,^18^, microelectromechanical systems (MEMS)^19^, textiles^20^, and self-deployable aerospace structures^21^. The original shape of thermally-induced SMPs will be deformed under an external stress at an elevated temperature above a transition temperature (e.g., glass transition temperature, T_g_ or melting temperature, T_m_)^22^. If the applied stress remains constant while the temperature is decreased below the transition temperature (T_g_ or T_m_), the SMP will maintain the temporary shape until it is heated above the transition temperature again. SMPs can be programmed into one shape per phase transition temperature, meaning a polymeric system with multiple transition temperatures will enable multiple programmed states^23^. Although SMPs possess advantages of low density, high elastic deformation, simple programming procedure and recovery control, the low recovery stress limits the size of components (i.e., the recovery stress of larger components is insufficient to counter their self-weight) when compared to other smart materials, especially shape memory alloys^7^. We aim to overcome this limitation by patterning lightweight, porous 3D architectures that have larger overall form factors than their bulk counterparts while still maintaining their shape memory characteristics.

Shape memory is impacted by chemical composition, microstructure, morphology, processing methods, and material layout. To further explore the practical applications of SMPs, a prerequisite is to precisely fabricate customizable architectures with controlled spatial distribution of the materials within a part or device. Three-dimensional (3D) printing is suitable for this purpose since it enables controlled patterning of materials into designed architectures unachievable using conventional methods. Many 3D printing techniques, such as fused deposition modeling (FDM) are confined to thermoplastic materials, which limits the temperature range over which parts can be used. Instead, thermoset materials generally exhibit relatively wider thermal usage range with improved mechanical properties. Previously, an inkjet-based commercial 3D printer^24^,^25^,^26^ was used to pattern SMP hinges in a foldable elastomeric structure using an ultraviolet (UV) photocurable thermoset resin. Another group has utilized UV curable photopolymers, acrylated polycaprolactone, to 3D print SMP devices using stereolithography^27^. However, it is difficult to ensure the resin fully cures using photochemical based processes, which can lead to poor aging behavior. An alternative method is an extrusion-based 3D filamentary printing technique, also known as direct ink writing (DIW), which employs highly concentrated, viscoelastic “inks” to assemble 3D structures by robotically extruding a continuous ink filament through a micro-nozzle at room temperature in a layer-by-layer scheme^28^. The prerequisite for this method is the necessity to design a gel-based viscoelastic ink possessing both shear thinning behavior to facilitate extrusion flow under pressure and a rapid pseudoplastic to dilatant recovery resulting in shape retention after deposition^29^,^30^,^31^,^32^. This 3D printing process has been used to produce fiber reinforced composites^33^, energetic materials^34^, cellular elastomeric architectures^35^, and graphene-based composites^36^. Although a number of ceramic, metallic, polymer and composite ink materials^28^,^37^ have been developed to fabricate various complex 3D structures, an example of this technique employed to create bio-based latent thermal cure 3D thermoset shape memory composite architectures has not been demonstrated.

In this work, we demonstrate a 3D printing strategy for the fabrication of shape memory structures with electrically and thermally responsive behavior at a broad range of temperatures using bio-originated composites based on novel cure chemistry. Our approach employs the precise deposition of composite filaments utilizing a pre-defined tool path to create complex structures. Two key challenges in this process are 1) the development of bio-based, long pot-life SMP composite inks that meet the printability requirements and 2) achieving the desired properties (e.g., mechanical, electrical and shape-memory properties) in the printed structures. To this end, we have developed novel conductive, thermoset composite ink and a patterning scheme that enables the manufacture of architecture-tunable structures possessing electrical conductivity, mechanical stiffness and shape memory effect. We have also demonstrated the application of this material to be post-processed into more complex shapes after an initial partial cure via origami methods. These 3D motifs can be used for a number of structural and functional applications, including stent-like devices and electrical interconnects and thermal sensors. Combining 3D printing with origami also provides a potential route for the creation of much higher complex 3D structures with defined material distributions and, hence, multi-stage thermal shape recovery profiles, which could enable SMP-based devices with unprecedented multifunctional performance. Increasingly this approach is being referred to as “4D printing” due to the extra dimension of time (or shape change) that such programmable materials add to conventional 3D printing and origami processes.

## Results

### 3D printing of bio-based thermoset shape memory composites

**Figure 1:**
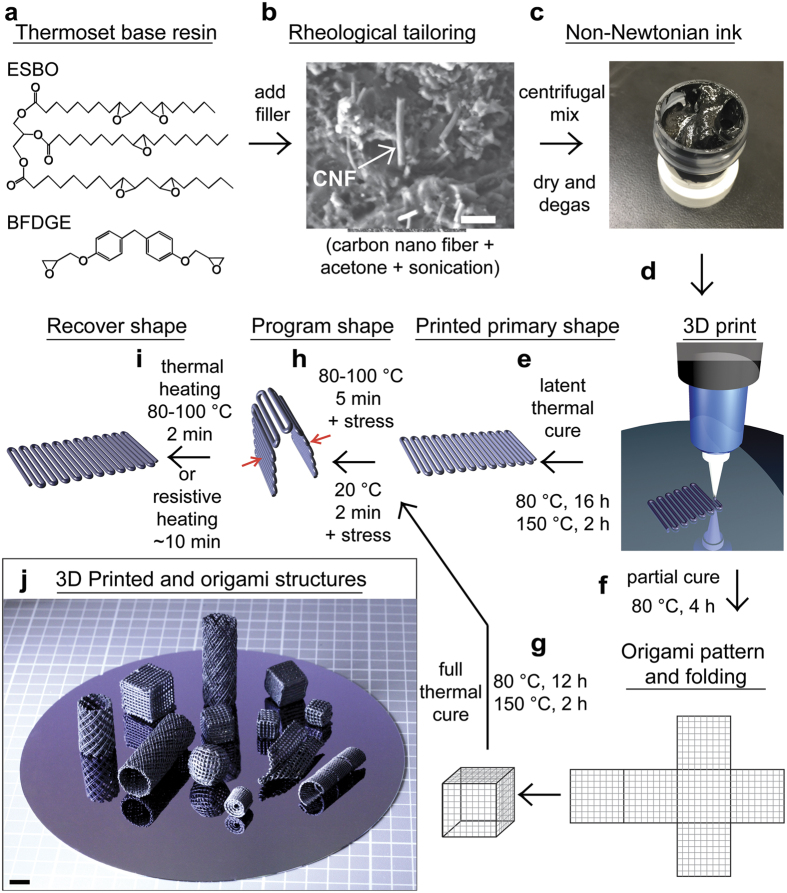
Ink development and fabrication process. (**a**) The chemical structures for epoxidized soybean oil (ESBO) and bisphenol F diglycidyl ether (BFDGE) starting materials used to prepare the base resin are shown. (**b**) Scanning electron micrograph of carbon nanofiber fillers, which are dispersed in acetone with sonication. The CNF suspension is added to the base resin with centrifugal mixing and acetone is removed resulting in the non-Newtonian inks shown in the optical image in (**c**). The ink then 3D printed through a micro nozzle to print complex 3D architectures as depicted in the schematic illustration in (**d**). At this point, two routes for final parts are available. Route 1 is shown in (**e**) where the printed part is thermally cured at 80 °C for 16 h, and then post cured at 150 °C for 2 h to obtain the printed primary shape. Alternatively, route 2 is shown in (**f**) where origami is used to fold the part after an initial partial cure at 80 °C for 4 h followed by (**g**) full curing of the folded part at 80 °C for 12 h with a post-cure at 150 °C for 2 h. The two routes converge at the programming step in (**h**) where part is heated to 80–100 °C for 5 min and programming is performed by applying an external stress to deform the part and maintaining the shape until the temperature is decreased to 20 °C for 2 min. Finally, the deformed part can recover to the original shape by thermal or resistive heating shown in (**i**). A range of 3D parts with complex geometries are shown in (**j**). The scale bar for (**b**) is 1 μm and for (**j**) is 1 cm.

The first task for this fabrication strategy is the development of room temperature printable SMP composite ink by tailoring the composition and rheology required for reliable flow through fine nozzles and shape retention after deposition. We also aim to create these inks from renewable feedstocks in order to ensure their availability and low-cost with minimal environmental impact. For this purpose, we chose epoxidized soybean oil (ESBO)^38^ as our starting material. ESBO is a commercially available material often used as a plasticizer for polyvinyl chloride (PVC). However, most materials made from ESBO are elastomeric and waxy in nature with poor mechanical properties^39^. To improve the mechanical strength of ESBO-derived materials, others have incorporated a more rigid co-polymer, such as bisphenol F diglycidyl ether (BFDGE) or bisphenol A diglycidyl ether (BADGE) into the base resin^40^. To make a SMP resin utilizing a renewable resource, we varied the concentration of ESBO and BFDGE, and added carbon nanofibers (CNFs) as fillers to tailor the mechanical properties and T_g_ while inducing electrical conductivity. The fabrication scheme for 3D printing and origami patterning of thermoset shape memory composites is illustrated in Fig. 1.

**Figure 2:**
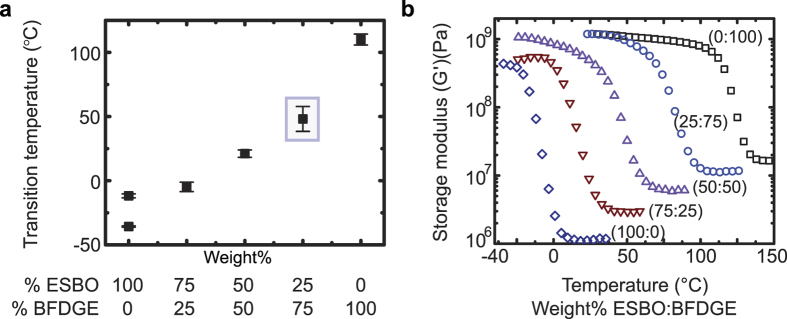
Thermal properties of base resin. (**a**) Glass transition temperature (T_g_) dependence on the ratio of ESBO: BFDGE found in the base resins (i.e., unfilled). The data point outlined in the box shows the base resin ratio (25:75 wt% ESBO: BFDGE) selected for formulation of CNF-filled inks. N = 5 per formulation. Error bars indicate standard deviation of the measurements. (**b**) Resultant storage modulus of the cured base resins from DMTA. Increasing amount of BFDGE in the ESBO: BFDGE ratio while decreasing ESBO resulted in an increase in the modulus.

We successfully cured all ratios of ESBO:BFDGE and they did not exhibit the previously reported waxy behavior, this was confirmed via differential scanning calorimetry (DSC) by the lack of an exothermic peak in the second heat after the initial cycle exposure to 150 °C. The 25 wt% BFDGE composites were tough elastomers, and with increasing concentrations of BFDGE the materials become even more rigid (Fig. 2a,b). The T_g_ measurements acquired from (DSC) and results from dynamic mechanical thermal analysis (DMTA) showed an increase in storage moduli and rubbery plateau exhibited with increasing amounts of BFDGE and help elucidate the nature of this effect. Figure 2a shows the trend that, as BFDGE concentration is increased in the base ESBO:BFDGE resin, the T_g_ increases accordingly, this is due to the rigid molecular structure and high T_g_ of BFDGE and flexible molecular structure and low T_g_ of the ESBO component. Previously, it was reported that fully cross-linked acrylated soybean oil exhibits two separate T_g_, which was also seen in the 100% ESBO (0% BFDGE) system^41^, and this explains the two data points shown in Fig. 2a at this concentration. The single T_g_ represented by the ESBO/BFDGE systems indicates a blend of the two monomers in the final polymeric structure. We selected the 25:75 wt% ESBO:BFDGE base resin ratio for ink formulation due to its non-elastomeric behavior and broad T_g_ that is above room temperature. Multiple transition shapes can be achieved in a shape memory polymer system when a broad T_g_ is present. Oscillatory cure studies and DSC data showed that 16 h at 80 °C with a 2 h post-cure at 150 °C was sufficient to fully cure this ink system (Fig. 4).

**Figure 3:**
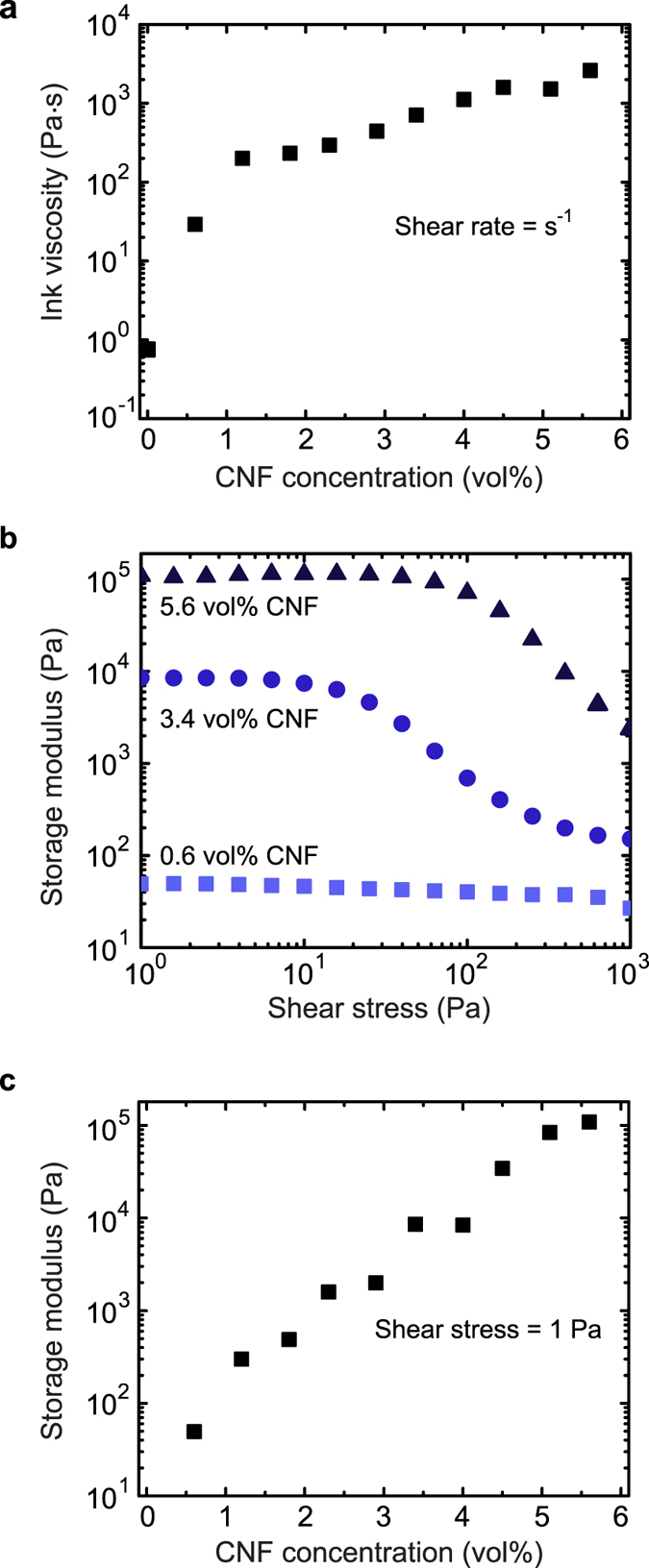
Rheological properties of composite inks. (**a**) Ink viscosity dependence on CNF concentration for viscosity at a shear rate of 1 s^−1^ showing shear thinning behavior. (**b**) Ink storage modulus as a function of shear stress for low, medium, and high CNF concentrations, which shows the onset of a yield stress at medium CNF concentrations. (**c**) Ink storage modulus dependence on CNF concentration for storage modulus at a shear stress of 1 Pa in the plateau regime. Increasing CNF concentration increases the ink storage modulus over 3 orders of magnitude.

**Figure 4:**
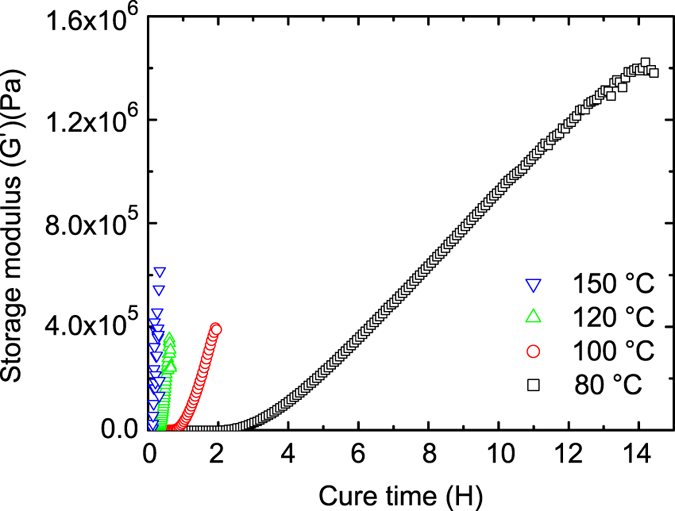
Cure temperature and time. The curing time decreases as the cure temperature is increased in this exothermic cure process. Less than 2 h is required to obtain a full cure at 150 °C. At 80 °C a better network is formed by the slower cure, as is shown by the higher resultant storage modulus, and was therefore used as the initial cure temperature of the systems. The curing time of the resins was determined by the maximum achieved storage modulus, at which point the experiment was stopped.

**Figure 5:**
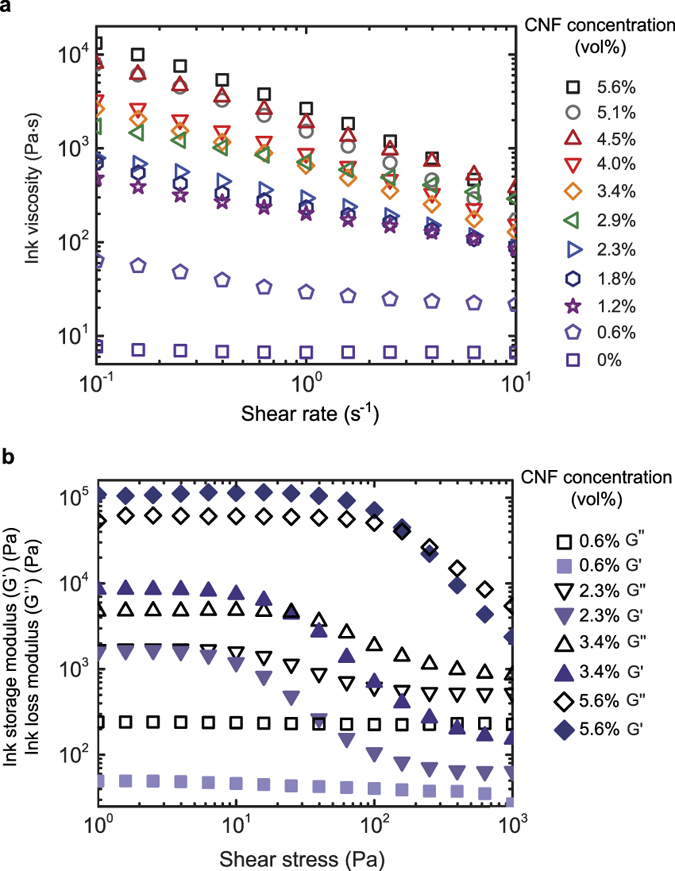
Ink viscosity as a function of shear rate and Ink storage and loss moduli as a function of shear stress at varying CNF concentration. **(a)** Addition of CNF results in shear thinning behavior with the effect more pronounced at higher concentrations, (**b**) At low CNF concentrations, the ink is liquid-like at all shear stresses (G′ < G″) (0.6 and 2.3% CNF). Addition of CNF filler at moderate concentrations results in the onset of a yield stress with solid-like behavior (G′ > G″) (3.4 and 5.6% CNF) at low shear stress and liquid-like behavior (G′ < G″) at high shear stress.

To modify the rheology and enhance the mechanical properties, CNF fillers were added to the base resin at varying concentrations. We found that increasing the amount of CNFs in the formulation increased the viscosity (Fig. 3a) while introducing a shear thinning behavior (Fig. 5). This effect ensures printability at low pressures, where shear forces are imparted to the ink while inside of the nozzle. Likewise, incorporation of CNFs increased the storage modulus (G′) and introduced a yield stress above a fiber filling threshold of ~3 vol% (Fig. 3b). Below 3vol% CNFs, we find that G′ is less than loss modulus (G″) at both low and high shear stress, which indicates liquid-like behavior under all measured stress conditions (Fig. 5). Above 3 vol% CNFs, G′ is greater than G″ at low stress, which is indicative of more solid-like behavior. However, at high shear stress for this same fiber filling concentration regime, G′ is less than G″ indicating the ink has shear thinned and flows. This behavior is important for shape retention for this room temperature printing process, as the ink can rapidly recover its more solid-like behavior after exiting the nozzle (i.e., in the absence of shear stress). Inks loaded with over 4 vol% CNF exhibit the shape retention behavior necessary for construction of 3D objects. However, 5.6 vol% CNF inks were chosen for their increased conductivity and ability to achieve shape memory actuation of (negative temperature coefficient polymer) 3D printed parts via resistive heating. In addition, the chemistry of these epoxy-based systems allows for a long pot life (printable up to 5 days at room temperature are possible after ink preparation), which means the rheology remains consistent throughout the print process and yields uniform structures (Supplementary video 1).

### Physical properties of the resultant resins

**Figure 6:**
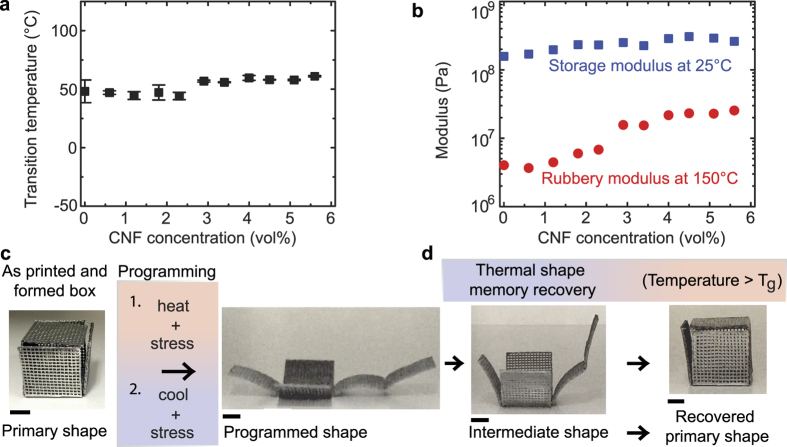
Thermal, mechanical, and shape memory properties of composite inks. (**a**) T_g_ dependence on CNF concentration in the 25:75 wt% ESBO: BFDGE base resin showing a small change at ~3 vol%. (**b**) Dependence of the cured material modulus on CNF concentration which shows a step change for the rubbery modulus at ~3 vol% CNF, indicated reaching percolation of the fiber network through the resin matrix. (**c**) A multi-layer, mesh-like structure is printed and then folded with origami to form a box, which is heated, flattened, and then cooled to form the programmed shape shown in (**d**). In (**e**), the part is heated to 80 °C and it goes through the intermediate shape transition before recovering the primary shape. All scale bars are 1 cm.

To elucidate the effect of CNFs addition on the properties of our SMP composites, we performed dynamic scanning calorimetry (DSC) and dynamic mechanical thermal analysis (DMTA) on neat and CNF-filled resins wherein the resin consisted of the 25:75 wt% ESBO:BFDGE ratio formulation for CNF filled systems and all ratios of ESBO:BFDGE. To ensure removal of residual thermal history, DSC measurements were taken after a second heating step to 150 °C. We find that addition of CNF results in a slight increase to the T_g_ (Fig. 6a) with a small step change at ~3 vol% CNF concentration. We postulate that this concentration corresponds to the onset of percolation of the CNF network within the resin matrix which both stiffens the material and increases thermal energy transport within the composite, resulting in a higher overall effective glass transition temperature. From DMTA measurements, we find that CNF addition slightly increases the storage modulus at room temperature (below the T_g_) and significantly increases the rubbery modulus at 150 °C (above the T_g_) (Fig. 6b). The storage modulus at room temperature ranges from ~150–300 MPa (a ~2-fold increase), whereas the rubbery modulus at 150 °C ranges from ~3–20 MPa (a ~6-fold increase) for the 25:75 wt% ESBO:BFDGE ratio formulation. Again, we observe a step change in the rubbery modulus at ~ 3 vol% CNF concentrations. This agrees with our previous postulation that percolation occurs at ~3 vol% since a percolating fiber network will have a more dominating effect on the overall mechanical properties when the matrix is rubbery rather than rigid.

In Fig. 6c, we demonstrate the ability to print a multi-layer, grid-like pattern with our CNF-filled SMP resins and fold the pattern into a complex structure (in this case a box) with an origami-like approach^42^,^43^. The material is then fully cured in that “primary shape”. Next, the structure is unfolded at a temperature above the T_g_ and then cooled below the T_g_ while maintaining the applied programming force that keeps the box unfolded, resulting in the “programmed shape.” To observe the shape memory effect, the box is heated above the T_g_ and the structure goes through intermediate shapes until it recovers its primary shape (i.e., the folded box) (Fig. 6c and Supplementary video 2 and S) due to the restoring forces from the polymeric chain network. These examples demonstrate the ability to tailor the transition temperature at which the parts recover, where Supplementary video 2 shows the recovery of a 75:25 ESBO:BFDGE structure that was programmed at a temperature below ambient conditions and supplementary video 3 shows the recovery of two origami structures at a temperature greater than ambient conditions (25:75 ESBO:BFDGE). The significance of varying the transition temperature of these materials via changing the ratios of ESBO: BFDGE affords the ability to print complex multi material parts that sequentially recover to their primary shape at different temperature ranges. Components of a complex part would be able to sequentially recover at different temperatures and allow for a stepwise and complex deployment of a device that has variable mechanical properties.

**Figure 7:**
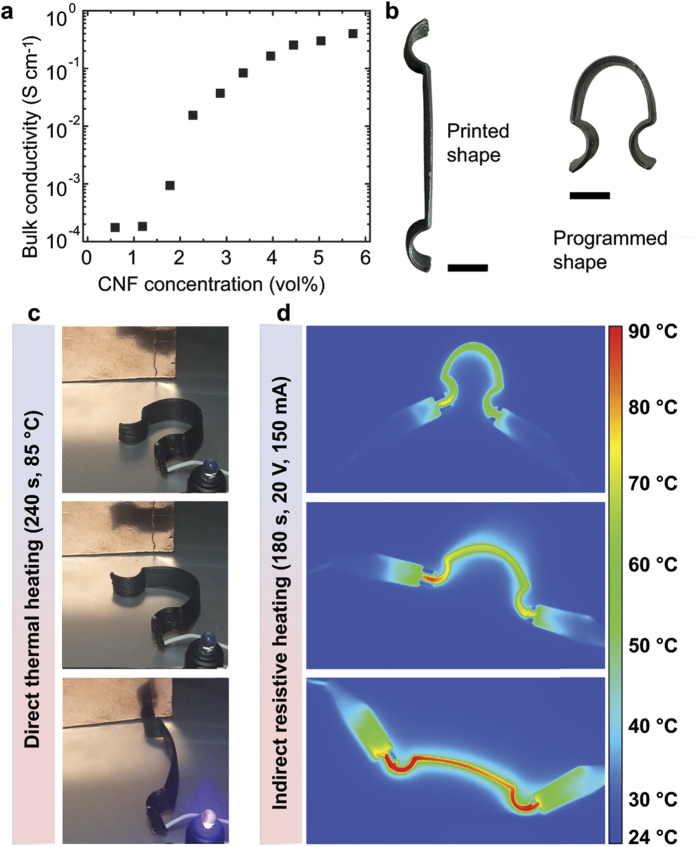
Electrical conductivity and multi-functionality of composite SMPs. (**a**) Plot of electrical conductivity dependence on CNF concentration where conductivity increases with increasing CNF concentration. (**b**) An electrically conductive (5.6 vol% CNF) part is shown in its as-printed or primary shape and its programmed shape. The part from (**b**) is connected to a power source and thermally actuated to change its shape and complete a circuit in (**c**) where an LED become powered and lights up after 240 s at 80 °C. (**d**) Thermal images obtained using an infrared camera are shown where resistive heating resulting from a power source of 20 V, 150 mA induces the shape change from the programmed shape to the printed shape after 180 s. In this case, the part continues to heat up and the shape change accelerates as the temperature increases. All scale bars are 1 cm.

**Figure 8:**
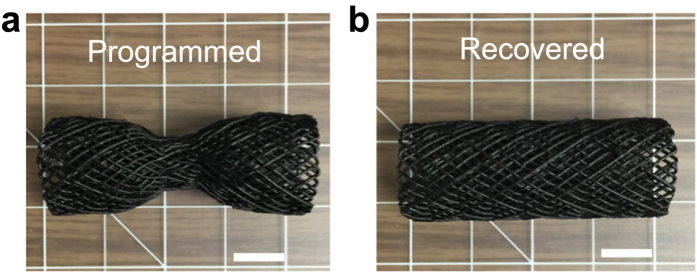
Programmed stent and recovered stent. (**a**) Demonstration of a (5.6 vol% CNF) 3D printed stent that has been programmed into a dumbbell shape. (**b**) Recovered cylindrical stent shape after direct thermal actuation at 85 °C. Scale bar is 1 cm in both panels.

Addition of CNF fillers has the effect of increasing electrical conductivity of our composite SMP materials. To quantify this effect, we measured the dependence of bulk electrical conductivity of our SMP composites with CNF concentration. We find that with increasing CNF concentration, the electrical conductivity increases over three orders of magnitude from ~0.0001 to ~0.4 S∙cm^−1^ when increasing the CNF concentration in our composite from 0.6–5.6 vol%, respectively (Fig. 7a). Due to the multifunctional nature of these novel printed SMPs, the shape change effect can be harnessed to complete an electrical circuit. For example, at high CNF concentration, the material is conductive enough to turn on low-power electronics such as a light emitting diode (LED). To demonstrate this effect, we printed and programmed the part shown in Fig. 7b with a 5.6 vol% CNF concentration ink. Next, we connected the programmed part to a 9 V power source and placed it in the vicinity of, but not in physical or electrical contact to, a copper electrode that was itself connected to a LED (Fig. 7c). The entire apparatus was located in an oven that was set to 85 °C. We monitored the configuration over time and found that, upon heating and given enough time to raise its temperature above the T_g_, the SMP device recovered its printed (or primary) shape and consequently completed the electrical circuit, which powered the LED (Fig. 7c and Supplemental video S4). Interestingly, resistively heating the SMP composite with an electrical stimulus can be used to induce the shape change. To demonstrate this effect, we again programmed the printed part and connected both ends to a 20 V, 150 mA power source to complete the circuit and we monitored it over time with an infrared camera (Fig. 7d). We found that after ~15 s, the part temperature is raised above the T_g_ and it begins to change shape. Over time, the material continues to resistively heat and the shape change is accelerated as the part temperature is raised (Supplementary video S5). After ~180 s, the shape recovery is complete and the final part temperature is ~90 °C. To demonstrate a potential application of this technology, we perform layer-by-layer printing of a mesh-like structure onto a cylindrical substrate using a printer equipped with a rotational axis (Supplemental video S6). Though not precisely the same size or shape, this architecture is designed to mimic the structure and behavior of a medical stent used to open occluded vasculature within the body. We program the stent into a partially compressed state and then heat it and observe a shape recovery into an open channel (Fig. 8 and Supplemental video S7).

## Conclusion

We present a general strategy for the fabrication of long pot-life, bio-derived thermoset SMP composites via 3D printing. Key factors for successful 3D printing of this composite material include rheological modification of the base SMP resin such that it not only serves as a printable ink for the 3D printing process to pattern designed architectures, but that it also exhibits shape memory. We printed and subsequently folded complex, 3D structures that exhibit both lightweight and good shape memory recovery. By varying the ratio of the individual components of the thermoset resin along with the addition of CNF fillers, we demonstrated how mechanical, thermal (T_g_) and conductive properties could be tuned. An application for such parts includes actuators or thermally responsive sensors. In addition to modifying the rheology and shape memory recovery, incorporation of conductive CNF filler also raised the electrical conductivity of the composite material. The shape morphing effect could be induced by direct thermal heating, which may be followed by some functional event, such as completion of an electrical circuit. Indirect, resistive heating could also activate shape memory recovery by the application of an electrical current through the material. We note that, as SMPs are continued to be developed, having 3D printing methods that enable fabrication of highly designed architectures should expand the range of applications where SMP composites can be utilized. In particular, our strategy emphasizes the possibility to explore the physical and mechanical property design space of in a systematic manner by modifying the resins combined with 3D printing and origami method advances (i.e., folding, twisting, and rolling).

## Methods

First, ESBO and BFDGE base resin is prepared by mixing these constituents at the appropriate stoichiometric ratios. Next, CNFs are dispersed in acetone by bath sonication and then added to the base resin to form a diluted suspension. The suspension is centrifugally mixed and acetone is removed, yielding a homogeneous, highly viscous, thixotropic ink that is suitable for our 3D printing process. The ink is then loaded into a syringe barrel and extruded through a micro-nozzle to pattern 3D structures (Supplemental video S1). After printing, structures are thermally cured at 80 °C for 16 h and then post-cured at 150 °C for 2 h. Alternatively, origami structures are obtained by a partial cure at 80 °C for 4 h prior to forming the desired structure. A final cure of the origami 3D structures is induced by 80 °C for the remaining 12 h and subsequent post cure at 150 °C for 2 h. The resultant part from either method can now be programmed into arbitrary temporarily shapes by heating it to 80~100 °C for 5 min and applying a stress to deform the sample (Fig. 1h) and maintain the shape until the temperature decreased to 20 °C for 2 min. This programmed part can recover to its original shape simply by resistive heating for 2 min or thermally heating to 80~100 °C again for 2–4 min.

### Base resin preparation

ESBO was mixed with BFDGE in controlled ratios to obtain the desired transition temperatures above ambient temperature. Epoxy resins were prepared using ESBO:BFDGE ratios of 100:0, 75:25, 50:50, 25:75, 0:100. Crosslinking of the ESBO and BFDGE resin was carried out through the use of a Lewis acid latent-cure catalyst added at 0.5 wt% to 1 ml of acetone. The prepared resins were degassed under vacuum over night to remove the acetone. Thermal curing was performed at 80 °C for 16 h with a 2 h post cure at 150 °C. From these formulations the 25:75 wt% ESBO:BFDGE resin was selected as the formulation for the base resin for the conductive SMPs composite based on the T_g_ of approximately 48 °C due to the transition being well above typical room temperature.

### SMP composite ink preparation

To make the ink printable, CNFs were added to the base resin in controlled concentrations. The CNFs are formed from graphitized cylindrical shaped platelets (length = 20–200 μm, diameter = 100 nm, MW of 12.01 g∙mol^−1^, density of 1.9 g∙ml^−1^ at 25 °C) were purchased from Sigma Aldrich. CNFs were first suspended in acetone with bath sonication (Branson B3510 ultrasonic cleaner) for 2 h. Next, CNF suspension was added to the base resin and mixed using a dual centrifugal mixer (DAC 150.1 FVZ-K Flacktek Speed mixer) at 3500 rpm for 1 min. The fiber-filled resin was heated at 50 °C and degassed with vacuum for 72 h to evaporate the acetone. Intermittent centrifugal mixing was performed to help agitate the mixture to aid in acetone removal.

### Ink rheology

Rheological properties of the ink were characterized using a stress-controlled Rheometer (AR 2000ex, TA) with a cone and plate geometry having a diameter of 40 mm and a cone angle of 2.006°. Temperature was held at 20 °C via Peltier plate temperature control of the bottom plate. Oscillatory stress sweeps at a frequency of 1 Hz from 10^−2^ to 10^3^ Pa were performed to record the G′ and G″ as a function of shear stress for inks with variable CNF concentrations. The yield stress (τ_y_) was defined as the stress where storage modulus falls to 90% of the plateau value. Continuous shear experiments were also performed to determine the apparent viscosity dependence upon shear rate for inks with variable CNF concentrations.

### Thermal and mechanical properties

Differential Scanning Calorimetry (DSC) was performed on the cured samples to determine the transition temperature of the composite materials using a Cryo-Discovery series DSC (TA Instruments). The samples were subjected to a heat, cool, heat cycle that ramped from 40–150 °C at 10 °C min^−1^, held at 150 °C for 1 min, ramped down to −120 °C at 10 °C min^−1^, and heated again at a rate of 10 °C min-1 up to 150 °C. Mechanical properties of the resultant materials were tested via dynamic mechanical thermal analysis (DMTA) on an Ares rheometer (TA Instruments) using a torsion cylinder adapter. Cylinders of the materials were cast in 1 ml syringes for testing. The cast rods of different CNF concentrations were tested by varying the temperature from 25–150 °C, at 1 °C min^−1^ at a frequency of 6.28 rad s^−1^.

### Electrical conductivity

Conductivity measurements were carried out using a four-point probe system (Jandel Engineering) with 1 mm spacing between probe points. Samples of different CNF concentrations were molded into thin disks of 20 mm in diameter and 2 mm in thickness. A size correction factor of 0.895 was used for the resistivity calculations. Five measurements were taken at each of four different locations near the center of the disks.

### 3D Printing

5.6 vol% CNF-filled ink was loaded into a 3 ml syringe barrel (EFD) attached by a luer-lok to a smooth-flow tapered nozzle (410 μm inner diameter). An air-powered fluid dispenser (Ultimus V, EFD) provided the requisite pressure to extrude the ink through the nozzle. The target patterns were printed using an *x-y-z* 3-axis positioning stage (ABL 9000, Aerotech). The 3D structures were printed onto fluoropolymer-coated glass slides, with a nozzle height of ~0.75 times the nozzle diameter to ensure moderate adhesion to the substrate and between printed layers.

### Thermal measurements

Thermal measurements were performed using a temperature-calibrated infrared camera (FLIR SC 660, FLIR systems) having 640 × 480 image resolution, 7.5–13 μm per pixel, while resistively heating the device with a power source set to 150 mA and 20 V. An image was acquired every second for a total record time of 180 s.

## Additional Information

**How to cite this article**: Rodriguez, J. N. *et al.* Shape-morphing composites with designed micro-architectures. *Sci. Rep.* **6**, 27933; doi: 10.1038/srep27933 (2016).

## Supplementary Material

Supplementary Video 1

Supplementary Video 2

Supplementary Video 3

Supplementary Video 4

Supplementary Video 5

Supplementary Video 6

Supplementary Video 7
